# Novel Small Molecules against Two Binding Sites of Wnt2 Protein as potential Drug Candidates for Colorectal Cancer: A Structure Based Virtual Screening Approach

**DOI:** 10.22037/ijpr.2019.15297.13037

**Published:** 2020

**Authors:** Hourieh Kalhor, Hamzeh Rahimi, Mohammad Reza Akbari Eidgahi, Ladan Teimoori-Toolabi

**Affiliations:** a *Department and Biotechnology Research Center, Semnan University of Medical Sciences, Semnan, Iran. *; b *Department of Molecular Medicine, Pasteur Institute of Iran, Tehran, Iran.*

**Keywords:** CRC, Molecular docking, Molecular dynamics simulations, Wnt signaling pathway, Wnt2 protein

## Abstract

Wnts are the major ligands responsible for activating Wnt signaling pathway through binding to Frizzled proteins (Fzd) as the receptors. Among these ligands, Wnt2 plays the main role in the tumorigenesis of several human cancers especially colorectal cancer (*CRC*). Therefore, it can be considered as a potential drug target. The aim of this study was to identify potential drug candidates against two binding sites of Wnt2. Structure-based virtual screening approaches were applied to identify compounds against binding sites of Wnt2 for inhibiting the interaction Wnt2 and Fzd receptors. The best hit compounds from molecular docking of National Cancer Institute diversity set II database were used for structural similarity search on ZINC database, obtaining large hit compounds query to perform a virtual screening and retrieving potential lead compounds. Eight lead compounds were selected while their binding affinity, binding modes interactions, and molecular dynamics simulations studies were assessed. Molecular docking studies showed that eight selected lead compounds can bind to the desired binding sites of Wnt2 in a high affinity manner. Bioavailability analysis of the selected lead compounds indicated that they possessed significant drug like properties. Thus, these lead compounds were considered as potential drug candidates for inhibiting Wnt signaling pathway through combining with the binding sites of Wnt2 and hindering the interaction of Wnt2 and Fzd receptors. Our findings suggest that Wnt2 binding sites may be a useful target for treatment for CRC fueling the future efforts for developing new compounds against Wnt signaling pathway.

## Introduction

Worldwide estimates show that colorectal cancer (CRC) is the third most common cancer and the second leading cause of death due to cancer (1). Its incidence in men is 30% to 40% while it is higher in men than in women (2). Despite using different regimens of chemotherapy for treating metastatic and non-metastatic CRCs, the failure rate is estimated to be about 90% (3). Wnt signaling pathway is over-activated in colorectal cancer cells (4). In vertebrates, Wnt signaling pathway can be activated by extracellular secretory proteins such as certain members of the Wnt family. Wnt ligands bind to Frizzled (Fzd) receptors and LRP5/6 co-receptors, which lead to the activation of canonical and non-canonical pathways (5, 6). Different studies have shown that among members of Wnt family (19 human Wnt proteins), Wnt2 protein (Wingless-type MMTV integration site family, member 2) has an important role in tumorigenesis of several human cancers including ovarian cancer, esophageal cancer, non-small-cell lung cancer, pancreatic cancer, gastrointestinal cancer, and CRC. Also, it was suggested that expression of Wnt2 in cancer cells is associated with the metastasis and tumor invasion (7-14). It is shown that Wnt2 as a crucial ligand complements Wnt/β-catenin signaling pathway activity in CRC while Wnt2 blocking destabilizes β-catenin protein in CRC cells (15). 

Despite clinical importance of Wnt2 in tumorigenesis, no inhibitor against this protein has yet been revealed. Because of its structural complexity, direct inhibiting of Wnt2 activity is very difficult. Three Dimensional structure of human Wnt2 has not ever been uncovered experimentally. However, only the crystal structure of the Xwnt8-mFzd8CRD has been reported, in which, Xwnt8 interacts with mFzd8CRD through two distinct sites (16). Detailed information about structure and function of the members of the Wnt family are poorly defined. In another study, structure of Wnt2 protein was investigated, by using protein structure prediction tools, molecular dynamics (MD) simulations, molecular docking, and energy analyses. Two binding sites 1 and 2 upon Wnt2 structure was identified in addition to the key residues involved in the binding of Wnt2 to Fzd receptor (Fzd7-CRD) (17). The residues of binding sites 1 and 2 are highly conserved in all Wnt ligands, these residues have the main role in the interaction with Fzd (18). It seems that the presence of both sites 1 and 2 are crucial for the perfect binding and activity of Wnts in Wnt signaling pathway. Therefore, these binding sites in Wnt proteins can be considered as important targets for the development of therapeutic agents. Currently, computer-aided drug discovery (CADD) approaches are widely applied in designing and development of new drugs (19, 20). In this approach, specific proteins which are upregulated in cancer cells or involved in protein-protein interactions (PPIs) are being targeted for drug discovery. Application of these *in-silico* methods before *in-vitro* experiments considerably saves the time and reduces the burden of chemical synthesis and biological testing for health systems (19, 21). Virtual screening is an essential computational technique for identification of new lead compounds from large database. There are two basic approaches for virtual screening namely ligand-based virtual screening (LBVS) and structure-based virtual screening (SBVS) (22, 23). 

In this study, a well-established set of computational methods were employed to find novel compounds inhibiting Wnt-Fzd interactions in Wnt signaling pathway. In order to start this strategy, NCI diversity set II database was employed to identify hit compounds. The structural similarity search of hit compounds was performed in ZINC database. Then, molecular docking was accomplished with new hit compounds from ZINC database. Finally, eight lead compounds were selected and their inhibitory characteristics and strength were analyzed and discussed. In order to improve potency and selectivity of lead compounds, their Lipinski’s rule of five was checked too.

## Experimental

*Selection and preparation protein*

 In SBVS method, drug discovery is carried out based on 3D structure of the desired protein while there is no previously synthesized inhibitory molecule as the leading sample (24). Therefore, identifying and preparing the target receptor is required in this method. Modeled Wnt2 structure in the previous study was used for molecular docking. Protein structure was prepared by removing water molecules using AutoDock Tools 4.2 (25). Subsequently atoms were adjusted to AutoDock atom types. Also, bond orders were assigned, and hydrogen atoms and Gasteiger-Marsili charges were added to the crystal structure. Afterwards, two different grid boxes were generated around the binding sites of Wnt2 with 1Å spacing between grid points with the dimension of 28 × 32 × 20 A°^3^ and 28 × 30 × 30 A°^3^ for the binding site 1 and 2, respectively.

*Library selection and preparation for docking*

Initially, a diverse chemical compound collection, National Cancer Institute (NCI) Diversity Set II, which is the starting point for the virtual screening in several drug design studies, was selected. These compounds in NCI Diversity Set II had been considered as the potential anti-cancer compounds which showed significant diversity in the chemical structure. 

*Virtual screening and identification of lead compounds*

The SBVS approach was used to find potential compounds to inhibit interaction of Wnt2 and Fzd receptors. Firstly, rigid docking of the binding sites of Wnt2 with NCI Set II Diversity library was performed by smina AutoDock (26). Docking results were ranked based on high binding affinity, two potential hits were separately constructed containing hit 1 against the binding site 1 and hit 2 against the binding site 2. Subsequently, structural similarity searches for both hits were performed separately in the ZINC database. Consequently, more extensive library was obtained for each hit. Afterwards, the libraries were filtered based on Lipinski’s rule of five to select compounds with drug likeness properties. Desired residues of the binding sites in the receptor treated as flexible and grid boxes were defined very specific though flexible docking that was carried out separately for each of libraries by smina AutoDock. The compounds showing the highest binding affinity were identified from each library as the lead compounds. Later, the drug likeness properties of the selected lead compounds were assessed to choose the potential compounds with the safe profile. 

*Optimization of lead compounds*

Many compounds may be known as the promiscuous compounds or pan assay interference compounds (PAINS). The PAINS possess many disruptive functional groups which can interact nonspecifically with the plenty of biological proteins and make false positive responses in high-throughput screening methods (27). In this regard, these PAINS compounds were removed using false positive remover software (http://cbligand.org/PAINS/search_struct.php). 

The bioavailability properties of the compounds are highly correlated with their chemical structures. These properties are one of the most important issues in rational drug discovery which helps the researchers to improve bioavailability properties of the candidate compounds. The physicochemical properties of the lead compounds such as molecular weight (MW), polar surface area (PSA), rotatable bonds (RB), the octanol–water partition coefficient (LogP), and solubility (LogS) were studied using OSIRIS Data Warrio software (28). Also, drug likeness score and toxicity risk parameters such as mutagenicity and tumorigenicity of the selected lead compounds were predicted.

*Molecular Dynamics simulations*

Molecular Dynamics (MD) simulations were performed by Gromacs package 5 of GROMOS53a6 force field (29), and the topology file of ligands was prepared for Gromacs using the PRODRG server (30). MD was run at 60ns for each protein-compound complex. The produced trajectories of MD simulations were applied for further analysis. 

*Visual presentations*

The structure of protein-compound complexes were visualized by PyMOL software, and hydrophobic contacts and hydrogen bonds between the compound(s) and Wnt2 protein were analyzed using Ligplot and discovery studio software (31-33). 

## Results

*Evaluation of the binding sites of the Wnt2 protein*

**Figure 1:**
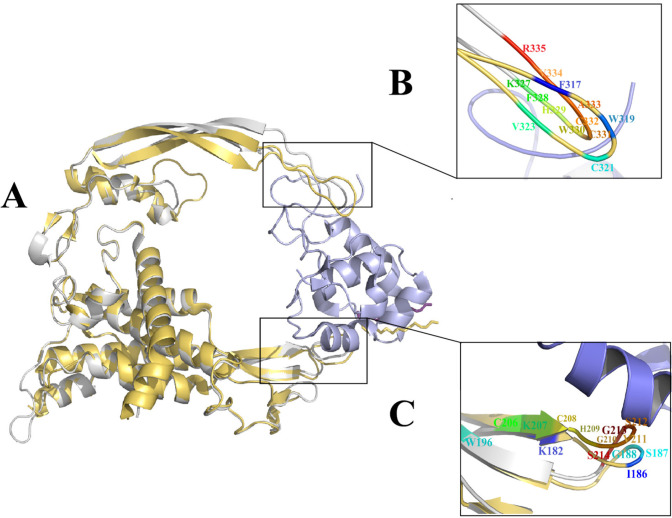
xWnt8-mFzd8 CRD and Wnt2 structure and evaluation of the binding sites on xWnt8 and Wnt2: (A) Structural alignment of the xWnt8 (yellow cartoon while Palmitoleic acid is shown sticks) and Wnt2 (gray cartoon while Palmitoleic acid is shown as purple sticks) in complex with mFzd8 CRD (light blue). (B) Close-up view of interacting residues of binding site 2 of xWnt8 and Wnt2 (C) Close-up view of interacting residues of binding site 1 of xWnt8 and Wnt2

The analysis of the crystal structure of the Xwnt8-mFzd8CRD revealed that Xwnt8 interacts with mFzd8CRD through two distinct sites. The first site contacts with mFzd8CRD through palmitoleic acid group and thumb loop amino acids (K^182^, I^186^, S^187^, G^188^ and W^196^). The second site forms a strong hydrophobic contact with mFzd8CRD through amino acids at the tip of index loop (F^317^, W^319^, C^321^ and V^323^) (16). Sumit Kumar *et al.* presented a mutational analysis of mWnt3a-mFz8CRD interaction. They reported that S^209^, G ^210^ (site 1), W ^333^ and V^337^ (site 2) residues play a key role in the binding to Fzd (34). In our previous study, 3D structure of Wnt2 was predicted and its binding sites and interaction with Fzd7 CRD was investigated in detail. Docking results displayed two distinct binding sites of Wnt2 including; C ^206^, K^207^, C^208^, H^209^, G^210^, V^211^, S^212^, G^213^, S^214^ (binding site 1), and K^327^, F^328^, H^329^, W^330^, C^331^, C^332, ^A^333^, V^334^, R^335^ (binding site 2) residues. In this study, superimposition of mWnt3a-mFz8CRD complex (PDBID:4F0A) and human predicted Wnt2 in complex with humanFzd7CRD (PDB ID: 5 T44) demonstrated that K^207 ^(K^182^ in Xwnt8), S^212 ^(S^187^ in Xwnt8), G^213 ^(G^188^ in Xwnt8) residues of binding site 1 and F^328 ^(F^317^ in Xwnt8), W^33 0 ^(W^319^ in Xwnt8), C^332^(C^321^ in Xwnt8), V^334 ^(V^323^ in Xwnt8) residues of binding site2 in Wnt2 protein are highly conserved, so these aforementioned residues were chosen as the main candidate residues for drug designing (Figure 1). 

*Structure-based virtual screening and identification of active compounds*

Rigid docking between the binding sites of Wnt2 and 1880 compounds from NCI Diversity Set II was performed by applying smina AutoDock. The results were sorted based on the highest binding affinity. Twenty-five compounds against the binding site 1 (hit 1) and the twenty-one compounds against the binding site 2 (hit 2) were chosen as two classes of potential hit compounds. These hits were used as the query for structural similarity search in ZINC database to design larger libraries. Cut off for this similarity search was 50%. Finally, 116704 and 71258 molecules were found for hit1 and hit2, respectively. These libraries were filtered using Lipinski’s rule of five to select the molecules showing drug like properties. It is well documented that orally active compounds follow Lipinski’s rule of five. The rules of five consisted of molecular weight <500, Xlogp (octanol-water partition coefficient) <5, H-bond acceptor <10, H-bond donors <5 (35). These filters reduced the size of libraries to 81122 and 36463 for hit1 and hit2, respectively. Subsequently, flexible docking with new hit compounds was performed, using smina AutoDock. Eventually, lead compound 1 and lead compound 2 comprising of 44 and 21 compounds were generated, respectively. 

*Evaluating bioavailability properties of lead compounds eased finding the best lead compounds*

**Figure 2:**
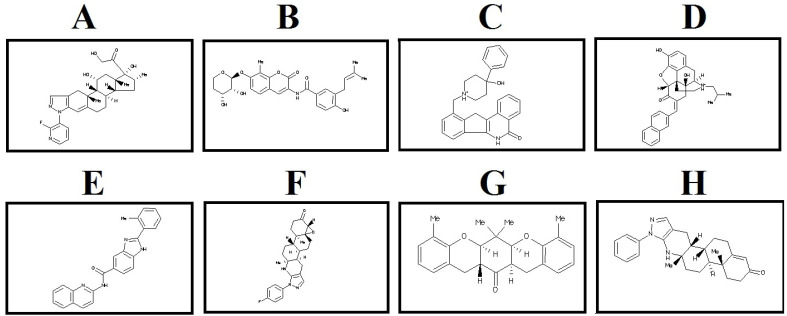
2D structure of the selected lead compounds from docking-based virtual screening. (A) ZINC40499329, (B) ZINC71316775, (C) ZINC35282053, (D) ZINC36221390 (E) ZINC66078286, (F) ZINC73408075, (G) ZINC60137214, (H) ZINC06482373

The selected lead compounds were filtered based on the physicochemical properties. Based on Data Warrior software user manual, compounds with cut off value of MW <500 Da, LogP <5, LogS <-6.5, H-bond donor <5, H-bond acceptor <10, TPSA ≤140 A^0^ and Rotatable bonds ≤10 can be considered as the promising drug candidates. LogP (the octanol–water partition coefficient) is a drug’s hydrophobicity property which is related to distribution and excretion of compounds. LogS (aqueous solubility) affect absorption and distribution of compounds. TPSA (topological polar surface area) is another parameter for determining the rate of absorption for each compound (36, 37). It is well documented that large molecules are less permeable because they have too many function groups and rotatable bonds. Drugs with high LogP have higher lipophilicity and lower absorption as well as higher metabolism, these drugs may bind to other hydrophobic macromolecules causing increased toxicity (38, 39). Almost, all selected compounds showed acceptable values for the evaluated parameters and all of them confirm drug like properties related to Lipinski’s rule of five (Table 1). Two dimensional structures of eight selected lead compounds are shown in Figure 2.

*Wnt2-compound complexes were stable during MD simulations*

**Figure 3:**
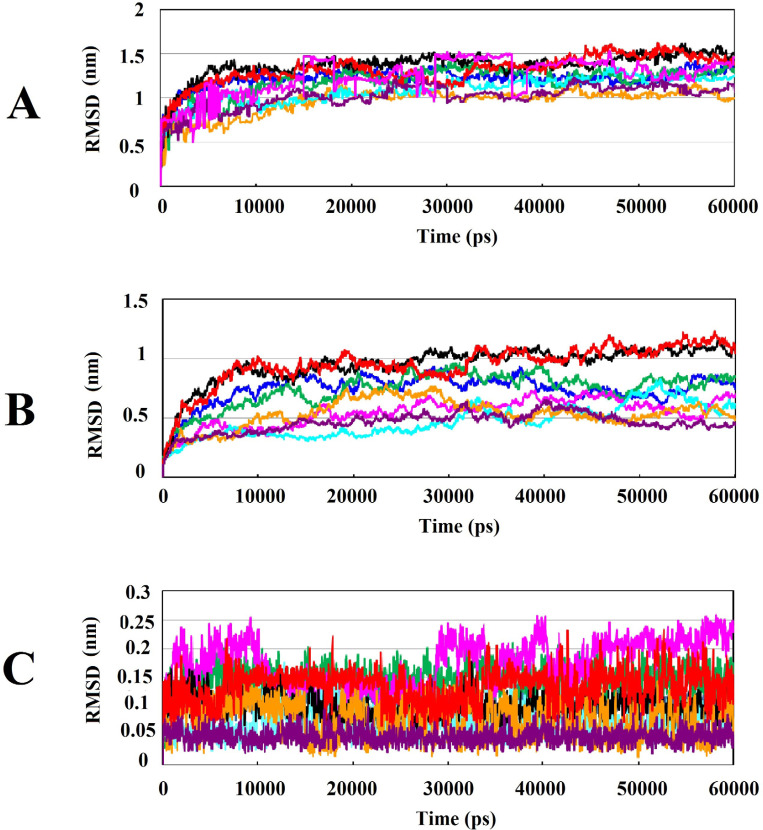
Analysis of RMSD value results during 60ns of simulations: (A) Root mean square deviation (RMSD) of backbone Cα atoms of the protein-compound complexes. (B) RMSD of backbone Cα atoms of Wnt2 in protein-compound complexes. (C) RMSD of backbone Cα atoms of compounds in protein-compound complexes. In all plots ZINC40499329, ZINC71316775, ZINC35282053, ZINC36221390, ZINC66078286, ZINC73408075, ZINC60137214, and ZINC06482373 are shown as black, green, blue, red, cyan, magenta, maroon and orang, respectively

Backbone Root Mean Square Deviation (RMSD) factor was used to investigate the conformational changes and system stability of Cα backbone of Wnt2 protein which was docked with the selected compounds in both complexed and non-complexed forms during 60ns of simulations. The calculated RMSDs of complexes are shown in Figure 3A; it can be seen that protein-compound complexes at the binding site 1 (ZINC40499329, ZINC71316775, ZINC35282053, ZINC36221390) had RMSD about 1.5 nm though ZINC36221390 showed the highest deviation, becoming stable in the last 20 ns. The RMSD values of protein-ligand complexes at binding site 2 (ZINC60137214, ZINC06482373, ZINC66078286) were practically similar; except ZINC73408075 which showed the highest deviation of RMSD. RMSD plots deciphered that the least RMSD value belongs to the protein-compound complexes at binding site 2. However, in both binding sites, the pattern of changes in RMSD in the last 20 ns was almost similar for all complexes reaching to stable values at this time. RMSDs of Wnt2 protein backbone in protein-compound complexes with respect to the initial conformation were investigated too, which was useful for studying the structural behavior and flexibility of the simulated system and estimating the protein stability. The highest RMSD values of Wnt2 were observed in ZINC40499329, ZINC36221390 (~1 nm) at the binding site 1, while the lowest RMSD values of Wnt2 were seen in ZINC60137214 (~0.5 nm) at the binding site 2. Interestingly, analysis of RMSD value of Wnt2 docked with ZINC73408075 showed lower deviation than its complexed form. Also it displayed similar structural behavior and flexibility to ZINC60137214, ZINC06482373 and ZINC66078286 (Figure 3B). To confirm the binding stability of the candidate compounds in the binding sites of Wnt2, RMSD values of each compound was generated and analyzed. During MD simulations, all compounds showed RMSD plots less than 0.17 nm and they were highly stable. Therefore, no considerable fluctuations were observed in the backbone of most candidate compounds, except ZINC73408075 which showed higher rate of continuos fluctuations (~0.15 – 0.25 nm) (Figure 3C).

*Hydrogen bonding and minimum distance between Wnt2 and selected lead compounds*

**Figure 4:**
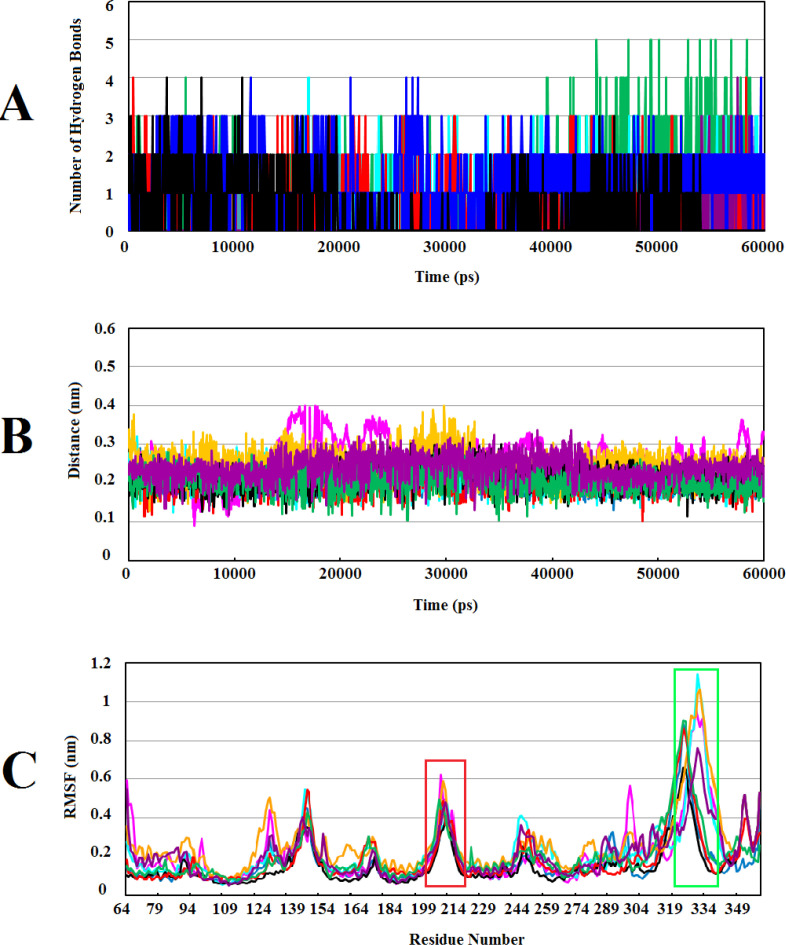
Calculating the number of H-bonds, the minimum distance, and structural fluctuations in Wnt2-compound complexes during 60ns of MD simulations: (A) The number of H-bonds between the Wnt2 and compounds. (B) The minimum distances between Wnt2 and compounds. (C) Root Mean Square Fluctuation (RMSF) of backbone Cα atoms of Wnt2 within complexes versus residue’s number in the sequence, the binding sites 1 and 2 are shown in boxes with red and light green, respectively. In all plots ZINC40499329, ZINC71316775, ZINC35282053, ZINC36221390, ZINC66078286, ZINC73408075, ZINC60137214, and ZINC06482373 are indicated as black, green, blue, red, cyan, magenta, maroon and orang, respectively

In order to have the knowledge about preservation of the binding affinity and stability between protein-compound complexes, H-bonds between molecules were analyzed through 60ns of MD simulations. This analysis showed that Wnt2-ZINC71316775 complex contains the highest (4–5) average H-bonds in the last 20ns of MD simulations whereas the least average H-bonds formed between Wnt2 and ZINC73408075 (0-1). The number of hydrogen bonds formed between Wnt2 and other complexes were 1 to 3. Also, the results showed that H-bonds formed between Wnt2 and ZINC35282053, ZINC40499329, ZINC66078286 and ZINC71316775 compounds were highly stable during MD simulations (Figure 4A). 

Minimum distance between two molecules can significantly affect their binding affinity. The strength of molecule interactions is dependent on the formation of interaction among polar functional groups and non-polar aromatic rings. Consequently, favorable interactions are seen at lower distances (40). The analysis showed that the minimum distances between Wnt2 and ZINC40499329, ZINC71316775, ZINC35282053, ZINC36221390, ZINC66078286, and ZINC60137214 compounds were ranged from ~0.15 to ~0.25 nm. In parallel, the minimum distance of ZINC73408075 and ZINC06482373 with Wnt2 were from ~0.1 to ~0.35 nm and ~0.3 to ~0.15 nm, respectively (Figure 4B). 

*The residues of Wnt2 binding sites showed the highest fluctuation during MD simulations*

Root Mean Square Fluctuation (RMSF) can obtain information about structural fluctuations during MD simulations. RMSF values were computed from the trajectories of each complex to assess the flexibility of individual residues (Figure 4C). Although residues in some complexes fluctuated more than the corresponding residues in the other complexes, overall patterns of RMSF in all complexes were very similar to each other. Assessing the residues in binding site 1 and 2 disclosed that the flexibility of these residues was increased in all complexes suggesting that these residues had mostly loop structures which were more flexible during MD simulations than other usual secondary structures. These flexible loops tend to be less rigid after binding to the compounds. 

*All selected protein–compound complexes possessed multiple hydrophobic bonds*

After docking the hit compounds to the binding sites of Wnt2, we calculated the threshold binding affinity from binding site 1 (-8 kcal/mol) and binding site 2 (-10 kcal/mol). Based on the cutoff, eight lead compounds were selected as the potential inhibitors for both binding sites of Wnt2. The Wnt2–compound complexes at binding site 2 had better binding affinity than binding site 1. The ZINC60137214 had the best binding affinity (-11.53 kcal/mol), while binding affinities of ZINC06482373, ZINC73408075, and ZINC66078286 were -11.44, -11.09 and -10.91 kcal/mol, respectively. As for binding site 1, binding affinity of ZINC40499329 (-9.77 kcal/mol) was better than the other compounds. ZINC71316775 was the second best and ZINC36221390 and ZINC35282053 followed that compound, with the binding affinities of -8.67, -8.48, and -8.515 kcal/mol, respectively. Wnt2-compound interactions were analyzed using Ligplot and discovery studio program, The significant Wnt2 protein residues involved in these interactions were I^151^, E^205^, C^206^, K^207^, C^208^, H^209^, G^210^, V^211^, S^212^, G^213^, S^214^, L^217^, W^221^ (binding site 1) and H^317^, V^318^, T^319^, R^320^, T^322^, C^326^, C^332^, K^327^, F^328^, H^329^, W^330^, C^331^, C^332, ^A^333^, V^334^, R^335^ (binding site 2) (Table 2). We found that hydrophobic interactions were more frequent than the other interactions; moreover the hydrogen bonds were considerable too.

## Discussion

Wnt signaling pathway can be considered as a good target for developing drugs in order to control various types of cancers. Although several attempts have been done for designing drug candidates against Wnt signaling pathway, just a few of them have been successful in the clinical stage (41).

Currently, several inhibitors have been detected which can target upstream or downstream proteins in this pathway leading to substantial anti-tumor activity. Porcupine inhibitors (IWPs) as small molecules inhibiting production of Wnt protein (42), FJ9 inhibiting β-Catenin-destruction complex, 3289–8625 and NSC668036 targeting the FZD and DVL-PDZ interaction are some examples of these compounds (43-45). Monoclonal antibodies such as OMP-18R5 and OMP- 54F28 have been developed in this regard (46). JW55 inhibiting PARP domain of tankyrase 1 and tankyrase 2, as well as JW67 and JW74 which suppress tumor growth in *Apc *mutant mice, have been introduced as drug candidates too (47, 48). Two most effective compounds, PFK115-584 and CGP049090 in a dose-dependent manner disrupt β-catenin/TCF complex (49). It is suggested that upstream targeting of Wnt signaling pathway can be effective too. Wnt signaling pathway activation is highly dependent on the binding of Wnt ligands to Fzd receptors (50, 51). It is well documented that Wnt ligands are the most important components of Wnt pathway. Blocking the binding of Wnt to Fzd receptor may suppress this pathway and increase apoptosis in cancer cells (15, 52). Despite the important role of Wnt proteins in Wnt signaling pathway, inhibitors which can directly inhibit Wnt proteins or block interaction of Wnt-Fzd have not yet been reported. Hammad *et al. *only designed small-molecule inhibitors against binging site 1 of Wnt4 (53). 

Studies have shown that Wnt2 protein is the main regulator of Wnt signaling pathway and its overexpression has been detected in various types of cancer, especially colorectal cancer (54-56). In this study, several compounds were identified against both binding sites of Wnt2 protein. MD simulations revealed that the compounds can efficiently bind and block Fzd-binding site of Wnt2.

**Figure 5:**
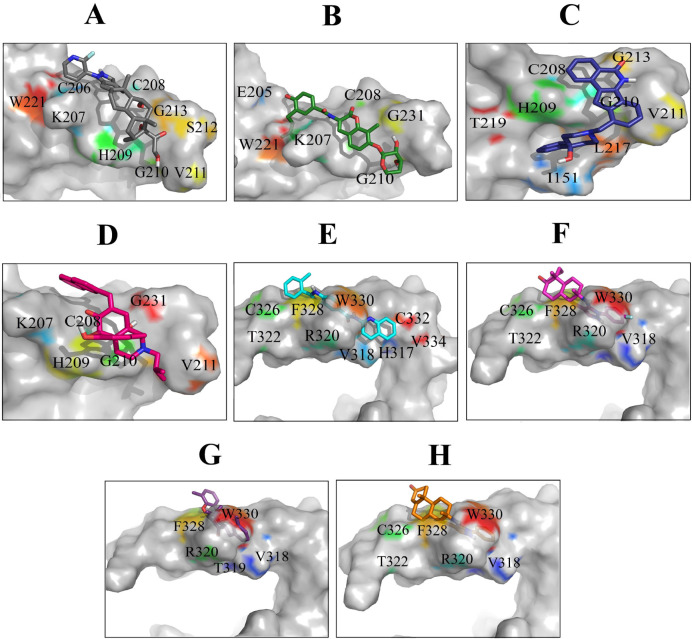
Binding orientations of residues within the binding sites of Wnt2 during interaction with the selected lead compounds: Interactions of binding site 1 of Wnt2 with (A) ZINC40499329, (B) ZINC71316775, (C) ZINC35282053, (D) ZINC36221390 and binding site 2 of Wnt2 with (E) ZINC66078286, (F) ZINC73408075, (G) ZINC60137214, (H) ZINC06482373

Most frequent protein–ligand atomic interactions are: hydrophobic interactions, hydrogen bonds, π-stacking and weak hydrogen bonds, salt bridges, amide stacking, and π- cation interactions (57, 58). These interactions are well known and extensively applied to drug design. Hydrophobic interactions are the key motivating force in protein–drug interactions (59). Therefore, lead compounds were investigated on the basis of the majority of the interactions with the binding sites of Wnt2 protein (Figure 5). The result of protein-compound complexes showed that these inhibitors not only can interact with the residues located in the binding sites but also can interact with some other residues such as I^151^, L^217^, E^205^, W^221^ (binding site 1) and V^318^, T^319^, R^320^, T^322^, C^326^, and C^332^ (binding site 2).

The most significant interactions between Wnt2 protein and the lead compounds were hydrophobic interactions. Hydrogen bonds were also contributed in the interactions between Wnt2 and compounds. The residues involved in hydrogen bonds were E^205^, C^206^, C^208^, H^209^, G^210^, V^211^ (binding site 1) and V^318^, R^320^ (binding site 2). In the binding site 1; ZINC40499329 compound revealed the highest binding affinity which was -9.7703 kcal/mol while its docking pose analysis showed four hydrogen bonds with C^206^, C^208^, G^210^ and G^213^ with the bond length of 2.68A˚, 2.48 A˚, 2.98 A˚, and 2.69 A˚, respectively. As well, ZINC60137214 compound in the binding site 2 showed the highest binding affinity of -11.53 kcal/mol. Different types of interactions; π- cation, hydrophobic interactions and a hydrogen bond with R320 residue with the bond length of 2.21A˚ were engaged in this binding which leads to the effective inhibition of Wnt2-Fzd receptor interaction. However, Wnt2-ZINC60137214 complex disclosed low RMSD which was almost stable in the last 30 ns. 

It is well documented that methyl (CH3) groups affect the biological activity or binding affinity of small molecules. The presence of methyl (CH3) group in the small molecules makes them more hydrophobic and more prone for binding to proteins. Commonly, the methyl (CH3) groups interact with a hydrophobic pockets in the protein binding sites (60, 61). Detailed analysis of the structure of ZINC71316775 and ZINC36221390 compounds against binding site 1 showed that they had a methyl (CH3) group which interacted with nonpolar residues of V^211^ and W^221^, respectively (Figures S1B and S1D, Supplementary File). Therefore, the presence of the methyl group in these compounds resulted in strong binding to Wnt2 protein. The RMSD evaluation of Wnt2-ZINC71316775 and Wnt2-ZINC36221390 complexes also confirmed that these compounds can efficiently bind to Wnt2. 

In year of 2009, more than 40% of the small molecules which are in the clinical trial phase or under development contained an aromatic ring. Aromatic groups are involved in weak interactions and playing a key role in determining fundamental chemical and biochemical properties. On the other hand, π- Cation interaction is a non-covalent molecular interaction between positively charged amino acid side-chains: arginine, lysine, histidine and aromatic ring of ligands (62). Arginine residue is more frequently involved in π-cation interactions than lysine side-chains (59). During analysis of protein-compound interactions, we found that the selected lead compounds against the binding site 2 contained benzene rings which had π-cation interaction with R^320^. Distances between R^320^ and π-orbital of ZINC66078286, ZINC73408075, ZINC60137214, and ZINC06482373 were 4.14 A˚, 3.63 A˚, 3.52 A˚, and 3.54 A˚, respectively (Figures S1E-S1H, Supplementary File). Also, these compounds showed higher binding affinity than the selected lead compounds against binding site 1. Previous studies have documented the role of the π-cation interactions in drug design. While Sorafenib which forms a π-cation interaction with the lysine residue of the human p38 MAP kinase was approved for the treatment of liver cancer, lysine residue of ErbB4 kinase having π-cation interaction with lapatinib was approved for the treatment of breast cancer (63, 64). Therefore, the selected lead compounds against binding site 2 can be potential drug candidates for inhibiting Wnt signaling pathway. 

Interestingly, we found that among selected lead compounds against binding site2, ZINC73408075 displayed different structural behavior and flexibility which was the highest fluctuation and instability in 40ns. Also, ZINC40499329 against binding site1 showed the highest RMSD value. Our investigation uncovered that they had a fluorine group in their structure. 

S. Alapour *et al.* have reported that the fluorine atom in the backbone of compounds generates higher non-conventional hydrogen bond interactions (inter and intra-molecular). These interactions probably can alter 3D structure of compounds and increase their flexibility (65). Fluorine as the small and most highly electronegative element can interact directly with the target protein or indirectly impress the polarity of other groups of the compound. This would lead to the improvement in the binding affinity of the compound to the target protein (66). Accordingly, ZINC40499329 and ZINC73408075 showed the highest fluctuations of RMSD in the free and complex form which may be due to the presence of fluorine atom upon phenyl ring (Figures S1A and S1F, Supplementary File). Also, they had high binding affinity to binding sites of Wnt2.

 It has been demonstrated that the compounds with even a single fluorine atom can remarkably play a role in medicinal chemistry. Nowadays, many fluorinated compounds are synthesized in the pharmaceutical studies which are widely used in the treatment of diseases such as different types of cancer (66, 67). As a result, ZINC73408075 and ZINC40499329 compounds can be promising drug candidates for inhibition of Wnt signaling pathway. 

RMSF value of a protein is a significant index of many biological processes such as macromolecular recognition, protein activity, and complex formations. Atomic fluctuations in Wnt2-compound complexes were evaluated by computing RMSF values of each complex throughout MD simulations. Studies have shown that higher fluctuations are related to the amino acids in the loop structures and surface-exposed regions in the protein-protein interactions (68, 69). S. Sikander Azam *et al.* reported that residues at the edges of NTD and CTD domains of Wnt4 protein (128–158, 201–241 and 272–351 residues) had loop structures and showed more fluctuations than the other residues which destabilize them during MD simulations (70). Accordingly, we found the highest fluctuations in the residues of binding site 1 and 2 (Figure 4C). It seems that these flexible regions of human Wnt2 may have an important role in the interaction with Fzd CRD receptors. In our previous study, we investigated conformational changes in xWnt8 bound to Fzd8CRD during MD simulations. Our analysis uncovered that binding sites of xWnt8 have the highest rate of fluctuations (17) . Based on these data and our results, theses flexible loop regions of binding sites of Wnt protein have a slight tendency to become rigid after binding to receptor or inhibitory compounds. 

Gane *et al.* demonstrated that the biological effects of compounds are associated with their chemical structure. Although the compounds with similar biological effects usually have identical structures, some compounds with dissimilar structures show comparable biological outcomes too (71). Interestingly, we realized that ZINC73408075 and ZINC06482373 compounds have identical structure and their interactions with the residues in binding site2 of Wnt2 were alike too (Figures S1F and S1H, Supplementary File). Surprisingly they showed different structural behavior and flexibility during MD simulations (Figures 3A and 3C). While alignment of their 2D structure disclosed that both have a similar core structure, the only difference was the presence fluorine atom which is attached to phenyl group in ZINC73408075. 

**Table 1.** Bioavailability properties of the selected lead compound

**TUM** ^j^	**MUT** ^i^	**DL** ^h^	**RB** ^g^	**PSA** ^f^	**HBD** ^e^	**HBA** ^d^	**CLS** ^c^	**CLP** ^b^	**MW** ^a^	**Compound Name**
BS1^*^										
NR	NR^***^	4.8029	3	108.74	3	7	-4.418	1.944	495.593	ZINC40499329
NR	NR	3.5929	6	134.55	4	9	-4.85	3.591	495.526	ZINC71316775
NR	NR	2.4803	3	53.77	3	4	-4.622	2.307	423.534	ZINC35282053
NR	NR	1.6555	3	71.2	3	5	-6.106	2.763	482.598	ZINC36221390
BS2^**^										
NR	NR	0.91752	3	70.67	2	5	-6.059	5.061	378.434	ZINC66078286
NR	NR	3.5501	1	59.45	1	5	-5.495	2.941	435.541	ZINC73408075
NR	NR	0.98722	0	35.53	0	3	-5.361	4.952	362.46	ZINC60137214
NR	NR	4.9035	1	46.92	1	4	-5.285	3.857	401.552	ZINC06482373

^a^Molecular weight of the molecule (g/mol); ^b^cLogP (lipophilicity); ^c^cLogS (solubility); ^d^H-bond donors; ^e^H-bond acceptors; ^f^Polar Surface Area (A2); ^g^Rotatable bonds; ^h^Druglikeness; ^i^Mutagenic; ^j^Tumorigenic; ^*^Binding site 1; ^**^Binding site 2; ^*** ^No risk.

**Table 2:** Binding affinity and analysis interactions between the selected lead compounds and binding sites of Wnt2

**Compound Name**	**binding affinity** **(kcal/mol)**	**HB-AAs** ^a^	**NH-AAs** ^b^
BS1^*^			
ZINC40499329	-9.77	C^206^ ,C^208^, G^210^,G^213^	K^207^, V^211^, H^209^,W^221^
ZINC71316775	-8.67	E^205^, G^213^	K^207^, C^208^, G^210^, W^221^
ZINC35282053	-8.48	V^211^	I^151^, C^208^, H^209^, G^210^, G^213^, L^217^, T^219^
ZINC36221390	-8.51	G^210^, H^209^	K^207^, C^208^, V^211^, G^213^
BS2^**^			
ZINC66078286	-10.91	___	H^317^, , R^320^, T^322^, C^326^ , W^330^, F^328^, C^332^, V^334^
ZINC73408075	-11.09	V^318^	R^320^, T^322^, C^32^, F^328^, W^330^
ZINC60137214	-11.53	R^320^	V^318^, T^319^, , F^328^, W^330^
ZINC06482373	-11.44	V^318^	R^320^, T^322^ , C^326^, F^328^, W^330^

^a^Hydrogen bonds forming Amino Acids, Hydrophobic bonds forming Amino Acids, ^*^Binding Site 1, ^**^ Binding Site 2.

## Conclusion

For the first time in this study, by using a combination of a well-established set of computational methods, eight inhibitory compounds were identified against binding sites in Wnt2. In parallel, based on physicochemical properties of compounds, binding affinity, binding modes, interactions of complexes and MD simulations analysis, we proposed that both binding sites of Wnt2 can be considered as a potential target for drug design. Combination of compounds against both binding sites, may be a reliable strategy for inhibiting Wnt signaling pathway which is activated in different types of cancer like colorectal cancer.

## Supporting Online Material

Supplementary Material
