# Supplementary material for: Novel Small Molecules against Two Binding Sites of Wnt2 Protein as potential Drug Candidates for Colorectal Cancer: A Structure Based Virtual Screening Approach
Source: Iran J Pharm Res. 2020 Spring;19(2):160–74. doi: 10.22037/ijpr.2019.15297.13037 (PMC7667561; doi:10.22037/ijpr.2019.15297.13037)
Supplement: Supplementary Material [file ijpr-19-160.s001.pdf]

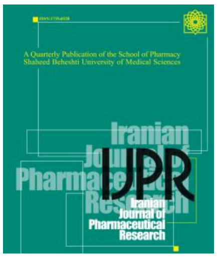

Supplementary Materials for

Novel Small Molecules against Two Binding Sites of Wnt2 Protein as potential Drug Candidates for Colorectal Cancer: A Structure Based Virtual Screening Approach

Hourieh Kalhor, Hamzeh Rahimi, Mohammad Reza Akbari Eidgahi* and

Ladan Teimoori-Toolabi*

*To whom correspondence should be addressed.

E-mail: mrakbari201177@yahoo.com; lteimoori@pasteur.ac.ir

Volume 19, Issue 2 (Spring 2020)

This PDF file includes:

Figure S1

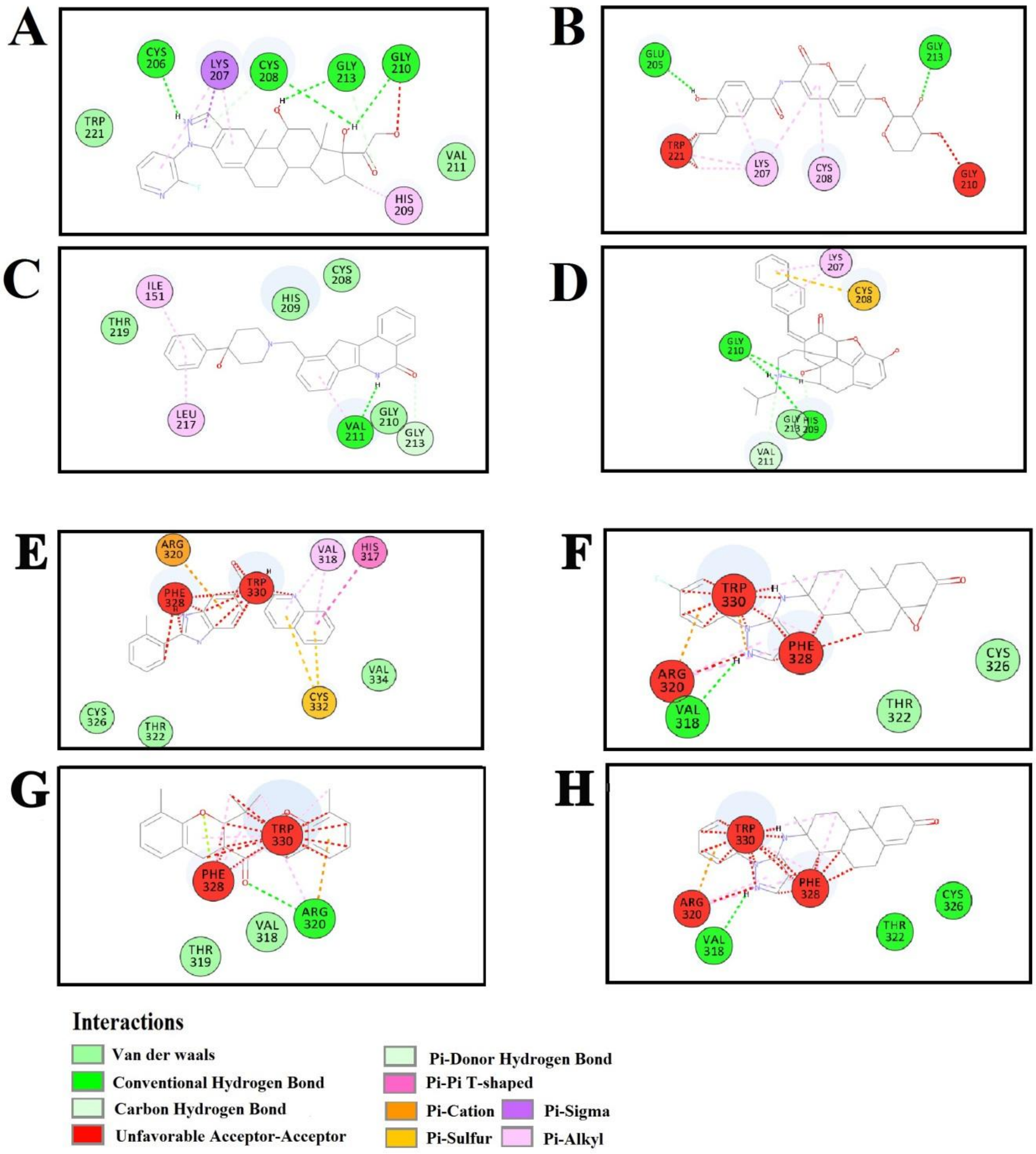

Figure S1. Detailed analysis of structure selected lead compounds and evaluation residue interactions between binding sites Wnt2 and these compounds: (A) ZINC40499329, (B) ZINC71316775, (C) ZINC35282053, (D) ZINC36221390 (E) ZINC66078286, (F) ZINC73408075, (G) ZINC60137214, (H) ZINC06482373.
